# “It Is Not the Robot Who Learns, It Is Me.” Treating Severe Dysgraphia Using Child–Robot Interaction

**DOI:** 10.3389/fpsyt.2021.596055

**Published:** 2021-02-23

**Authors:** Thomas Gargot, Thibault Asselborn, Ingrid Zammouri, Julie Brunelle, Wafa Johal, Pierre Dillenbourg, Dominique Archambault, Mohamed Chetouani, David Cohen, Salvatore M. Anzalone

**Affiliations:** ^1^Department of Child and Adolescent Psychiatry, APHP, Sorbonne Université, Hôpital de la Pitié-Salpêtrière-Charles Foix, Assistance Publique Hôpitaux de Paris, Paris, France; ^2^CHART EA 4004, THIM, Paris 8 University, Saint Denis, France; ^3^ISIR, Sorbonne Université, CNRS UMR 7222, Paris, France; ^4^Computer Human Interaction in Learning and Instruction Lab, Ecole Polytechnique Fédérale de Lausanne, Route Cantonale, Lausanne, Switzerland; ^5^Department of computer Science and Engineering, University of New South Wales, Sydney, NSW, Australia

**Keywords:** human-robot interaction, handwriting, serious-game, occupational therapy, dysgraphia, learning-by-teaching

## Abstract

Writing disorders are frequent and impairing. However, social robots may help to improve children's motivation and to propose enjoyable and tailored activities. Here, we have used the *Co-writer* scenario in which a child is asked to teach a robot how to write via demonstration on a tablet, combined with a series of games we developed to train specifically pressure, tilt, speed, and letter liaison controls. This setup was proposed to a 10-year-old boy with a complex neurodevelopmental disorder combining phonological disorder, attention deficit/hyperactivity disorder, dyslexia, and developmental coordination disorder with severe dysgraphia. Writing impairments were severe and limited his participation in classroom activities despite 2 years of specific support in school and professional speech and motor remediation. We implemented the setup during his occupational therapy for 20 consecutive weekly sessions. We found that his motivation was restored; avoidance behaviors disappeared both during sessions and at school; handwriting quality and posture improved dramatically. In conclusion, treating dysgraphia using child–robot interaction is feasible and improves writing. Larger clinical studies are required to confirm that children with dysgraphia could benefit from this setup.

## Summary

Using a longitudinal single-case study design, we show that treating dysgraphia using child–robot interaction combining a learning-by-teaching scenario and gaming is feasible and improves writing.

## Introduction

Handwriting is important for education. It is a complex perceptual–motor task as it involves attention, perceptual, linguistic, and fine motor skills (1, 2). When writing acquisition becomes challenging, it can lead to dysgraphia, defined as impairment in quality or speed to achieve sufficient smooth and automatized handwriting according to age. Dysgraphia is not a disorder *per se* but a specifier of neurodevelopmental disorders (NDDs) such as attention deficit/hyperactivity disorder (ADHD), developmental coordination disorder (DCD), dyslexia, and autism spectrum disorder (ASD) (3). In addition to the specific tasks involved in handwriting acquisition, when a child has dysgraphia, it can be negatively reinforced by avoiding writing due to anxiety that limits the improvement of the writing. This avoidance also limits writing opportunities that are compulsory in a training process (4, 5). When difficulties are detected, they are usually addressed by occupational therapists. Occupational therapy may be provided to the child (6), or consultation may be provided to the teacher (7). In addition, the approach taken may focus on remediating potential causes of handwriting problems (e.g., attention deficit that impacts handwriting automatization) or handwriting itself (2). When it focuses on handwriting, remediation proposes pen-and-paper exercises aimed at automatizing the writing process by doing geometrical figures, letters, and finally words and sentences (8). These exercises are very close to tasks carried out in school and can be a challenge for many children. Some children with dysgraphia express frustration—sometimes refusal—regarding treatment sessions. However, systematic reviews have shown that rehabilitation of writing including handwriting practice, relaxation, or sensory-based training are efficient approaches and are recommended. Regardless of treatment type, efficient interventions include handwriting practice and more intensive treatment (e.g., ≥2 sessions/week; ≥20 sessions of total duration) (6). For instance, the Cognitive Orientation to Occupational Performance (CO-OP) program aims to facilitate the planning of movements of children with DCD and handwriting difficulties (9). The “handwriting without tears” program is a developmentally and multisensory based handwriting curriculum that aims to promote appropriate practice by using stages from imitation to copying to independent writing (10). To limit the disability induced by difficulty in handwriting, some adjustments can be proposed. In a school context, it is important to train teachers to favor oral presentations or propose photocopies of lessons for children with the more severe difficulties and to avoid double tasks and cognitive overload. It is at the moment impossible to predict which kind of children will benefit from rehabilitation and which of them will need assistive technologies (e.g., use of computer for all writing tasks) for compensation (2). Information and communication technologies (ICTs) have opened new ways to help people with NDD. These technologies allow the creation of real-life situations in a controlled area and offer clinicians and educators different supports to work with (11). ICT-based interventions include (1) smartphone and tablet apps that aim to facilitate specific aspects of daily life; (2) serious games that can be described as “digital games and equipment with an agenda of educational design and beyond entertainment” (12); (3) robots in the context of specific training scenarios (13, 14).

ICT have been used in both clinical and educational/home settings. In education, children with handwriting problems are considered children with special needs (CSNs). In clinical practice, children are seen as having an NDD associated with dysgraphia. Traditionally, the approaches to ICTs in education have been divided into “Learning about ICTs” and “Learning with ICTs,” in other words, between “Education in ICTs” and “ICTs for Education.” The former approach concerns technical, robotics-oriented education, while the latter implies teaching different subjects (technical and nontechnical) through ICTs. For example, in the case of robotics, it is of paramount importance to distinguish between “ICTs used for CSN” and “ICTs used by CSN” (15). Most studies in the field ICT and learning regard (1) reading, spelling, math, and writing acquisition when they come from education [e.g., 17, 18] or (2) ASD when they come from child psychiatry (16). As said previously in the field of handwriting, ICT has been first used to compensate the consequences of handwriting in terms of quality or speed through the use of computers and computer software (2). A second application of ICTs as assistive technologies is to combine word processing software on laptops and speech recognition software during classroom. This method improves school inclusion of children with severe writing difficulties. However, they can impose an additional burden in terms of working memory and requires additional training to be used fluently (17).

With the improvements of ICTs in terms of sensors, processing speed, and algorithms, recent ICT applications in the field of NDD and handwriting include the use of electronic sensors (e.g., tablets, 2D and 3D camera) and algorithms to help characterize movement impairments or dysfunction in NDD (18). Specifically, ICT has been used to characterize motor kinematics and developmental characteristics during writing acquisition (19). Some new features from computing recordings have been defined such as signal-to-noise velocity peaks difference (20). Using principal component analysis, Asselborn et al. (21) defined three independent dimensions and four computerized scores related to kinematics, pressure, pen tilt, and static features to characterize dysgraphia. Several authors developed machine learning methods to diagnose children with dysgraphia based on handwriting on tablets (22–24). To our knowledge, the use of ICTs in a treatment perspective is very limited. The first exploratory controlled trial suggests that computer-assisted instruction treatment is efficient (25). The second preliminary exploratory study showed promising results with a robot-assisted handwriting activity. Authors used an iterative design and evaluation protocol to define a robot-assisted handwriting activity related to the shape and the dynamics of the letters. Their scenario combined specific computerized instructions and pupil–robot interaction through haptic properties of the robotic platform Cellulo to offer interactive feedbacks (26). Here, we present (1) the *Co-writer* setup that combines several modules (a Wacom tablet, a Nao-robot, a 2D camera to assess posture, specific metrics to assess writing, and a platform of serious games–*Dynamico*) in the context of a learning-by-teaching scenario (27); (2) how we used this setup to reopen handwriting therapy in a child with complex NDD associated with dysgraphia and refusal to write.

### Patient's Characteristics Prior Training

R was an 8-year-old boy when he was assessed for severe dysgraphia and refusal to write at school. In the past, he tried to break his pen during writing due to frustration and anger, and he needed to repeat his first grade because of lack of writing acquisition (a practice tolerated in France). His parents divorced when R was 3 years old. Family history showed that R's father and mother both had dyslexia, and R's mother had postnatal depression. Personal history included a week of postnatal hospitalization following delivery with forceps and ventilation mask. Apgar scores were 3, 8, 9, and 10. Weight at birth was 2,975 g with normal cranial perimeter and size. R's early development was marked by psychomotor agitation. He received physiotherapy at age 1 year. He started to walk at 13 months, but walking was very unstable with a lot of falls. Oral language was subnormal but R had early phonological impairments, and he was not understandable when speaking in kindergarten. At age 5 years, he entered a classroom with special education. At age 6 years, he received a diagnosis of ADHD, and treatment with methylphenidate (30 mg/day) began. When R was admitted to our department, we conducted an in-depth assessment summarized in Table 1. He was diagnosed with ADHD, severe dyslexia, and DCD with severe dysgraphia that were impairing for schooling. At age 8, he was refusing to use any kind of pens.

**Table 1:** Patient's characteristics.

**Assessment: age**	**Results**	**Comments**
Wechsler Intelligence Scale for Children (WISC IV): 7 years	Verbal Comprehension Index: 120	Agitation was important during testing. The cognitive evaluation shows heterogeneous abilities. Verbal abilities were excellent. Attention was poor especially for memory task.
	Perceptual Reasoning Index: 107	
	Working Memory Index: 73	
	Processing Speed Index: 109	
Autism diagnostic Interview-Revised: 5 years	Social interactions: 9 (threshold = 10)	R had subliminal social difficulties and significant repetitive behaviors. Diagnosis of autism was not retained at age 8 from direct assessment.
	Verbal communication: 6 (threshold = 8)	
	Stereotyped behaviors: 7 (threshold = 3)	
	Development score: 4 (threshold = 1)	
Language assessment (Age 7 and 8 months)	**Oral language**	Severe phonological disorder but good other oral language abilities
	Phonology: all scores are pathological (−1.9 to −6.8 SD) from mean	
	Lexicon reception: all scores in the average range.	Severe dyslexia that could only be assessed using tasks for the first trimester of the first grade (6 years in France) in which all scores ranged between 6 and 30 percentiles.
	Lexicon expression: normal score for concrete vocabulary, −2 SD for abstract lexicon	
	Syntax reception: all scores in the average range.	
	Syntax expression: all scores in the average range (−1 SD)	
	**Written language**	
	Reading acquisition has not yet started and assessment is impossible	
Language assessment (Age 10)	*Written language* using reading tests based on second grade (6 years in France)	Severe dyslexia remains. R has entered in the mechanism of reading but with a large delay compared to his age group.
	Word identification score of non-words is −3.5 SD, of regular words is −2.8 SD, of irregular words is < -3 SD.	Despite this delay, some comprehension of written text was possible as +0.9 SD of second grade was the average of third grade.
	Reading text is painful with time at−0.4 SD, number of errors at−3.5 SD but a comprehension score at +0.9 SD	
Motor Battery Assessment: 8 years	Degradation score = 24	Hypotony was obvious and R had difficulties in motor control. Manual dexterity was difficult. R needed to stop his breathing to focus correctly. The grasping of the pen was hypotonic and the pen fell many times. R was right-handed.
	<1st percentile	
	−4.23 standard deviation from mean	
Writing BHK: 8 years	The BHK could not be scored because of too poor quality. (max score = 65)	R. could not write in cursive letters. He wrote some capital letters. The movement was chaotic like he was throwing the pen. Some letters were impossible to read.
Writing BHK: 9 years	The BHK could not be scored because of too poor quality (max score = 65)	The second testing remained very challenging. Only the 5 first lines were realized and R wrote only in capitals instead of cursive. The size of the letters was very large.

In addition to methylphenidate, R received remediation sessions with a reading specialist and was admitted to our special school for multidimensionally impaired children (28). Given the severity of DCD and dysgraphia, R also started specific remediation for writing every week (40-min session) with an occupational therapist. The therapist was limited in R's remediation, since he was complaining about writing. The sessions were anxiogenic; he tried to break his pencil when frustrated. The training was progressive to help the child to improve self-confidence and avoid learned helplessness. However, after 1 year, the validated testing [brave hand writing kinder (BHK), see *Method*] was still impossible to score and R refused to use a pencil in classroom. We therefore discussed with R and his parents to train handwriting with the *Co-writer* setup.

## Method

### *Co-Writer* Setup

**Figure 1:**
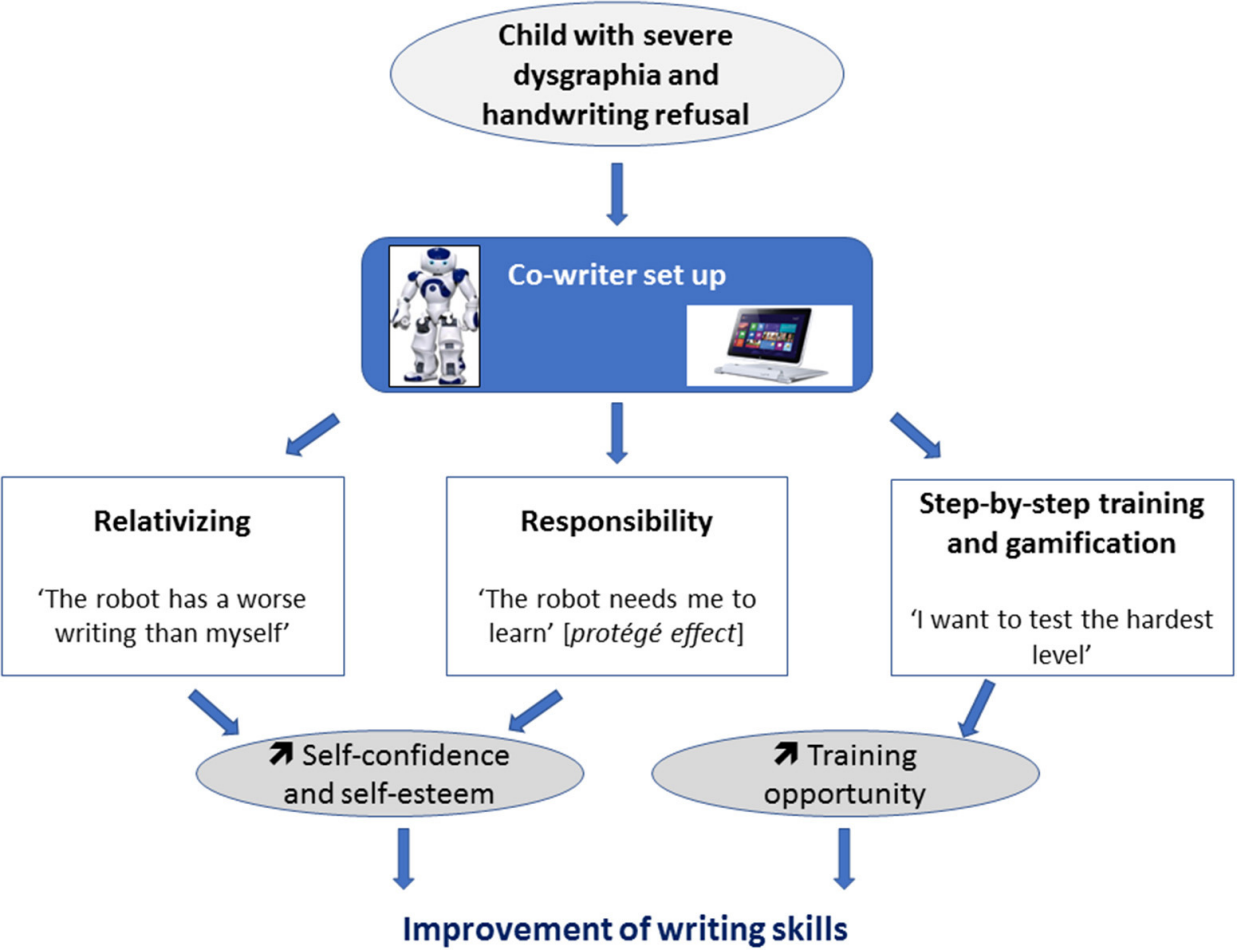
Cognitive and affective processes and functional training involved in the *Co-writer* setup.

The *Co-writer* setup was built in order to combine functional training and cognitive/affective processes during remediation (Figure 1). The goal was to stimulate in parallel relativizing and responsibility, on the one hand, and handwriting training, on the other hand. The global architecture of the setup is detailed in a video demo summarizing the 20 sessions available at https://youtu.be/0iLScP0PjzU. The first component of the setup is a software that allows the extraction of handwriting automatic features (static, kinematic, tilt, and pressure) from a computer tablet during writing. The Wacom tablet (Wacom Cintiq pro) allows the extraction of the pen's position (*x, y*), the pen tilt in two axes, as well as the pressure between the pen and the surface of the tablet. The sampling frequency of the tablet can go up to 200 times per second (Hz). Features have been detailed in Asselborn et al. (23).

The second component is a robotic platform Nao that remains beside the child. We previously showed that participants' engagement was better with a physical robot than an avatar (29). During sessions, the child writes with a stylus on a Wacom Cintiq Pro connected to a laptop. Ubuntu was installed on the laptop with the Cowriter software (30). We asked the child to teach Nao how to write. One after another, Nao pretends to write on the Wacom tablet by moving its arm, and the child writes on the tablet to correct the writing of the robot. The cowriter research project aims to help children with difficulties using an original approach: the child plays the role of the teacher and the robot acts as a student requiring help to improve its handwriting. This approach is called *learning by teaching* and has several advantages. First, it brings a positive reinforcement of the child's self-esteem as he/she becomes the one who “knows and teaches” and no longer the worst student in the classroom (31). Second, we can observe a huge gain of motivation as the child, feeling responsible for the robot, is committed to the task with an intensiveness way higher compared to when practicing in a normal environment. This particular interaction where children feel responsible for the robot is called the *protégé effect* (27). Various researches have shown that learning with a physical robot can be more efficient than learning from a more classical approach (32, 33). We hypothesized that this setup could be more engaging for the patient than a classical pen-and-paper remediation. Furthermore, one of the best drivers of training is evaluation (34). The teaching procedure is one of the more obvious situations during which one needs to evaluate its own abilities.

**Figure 2:**
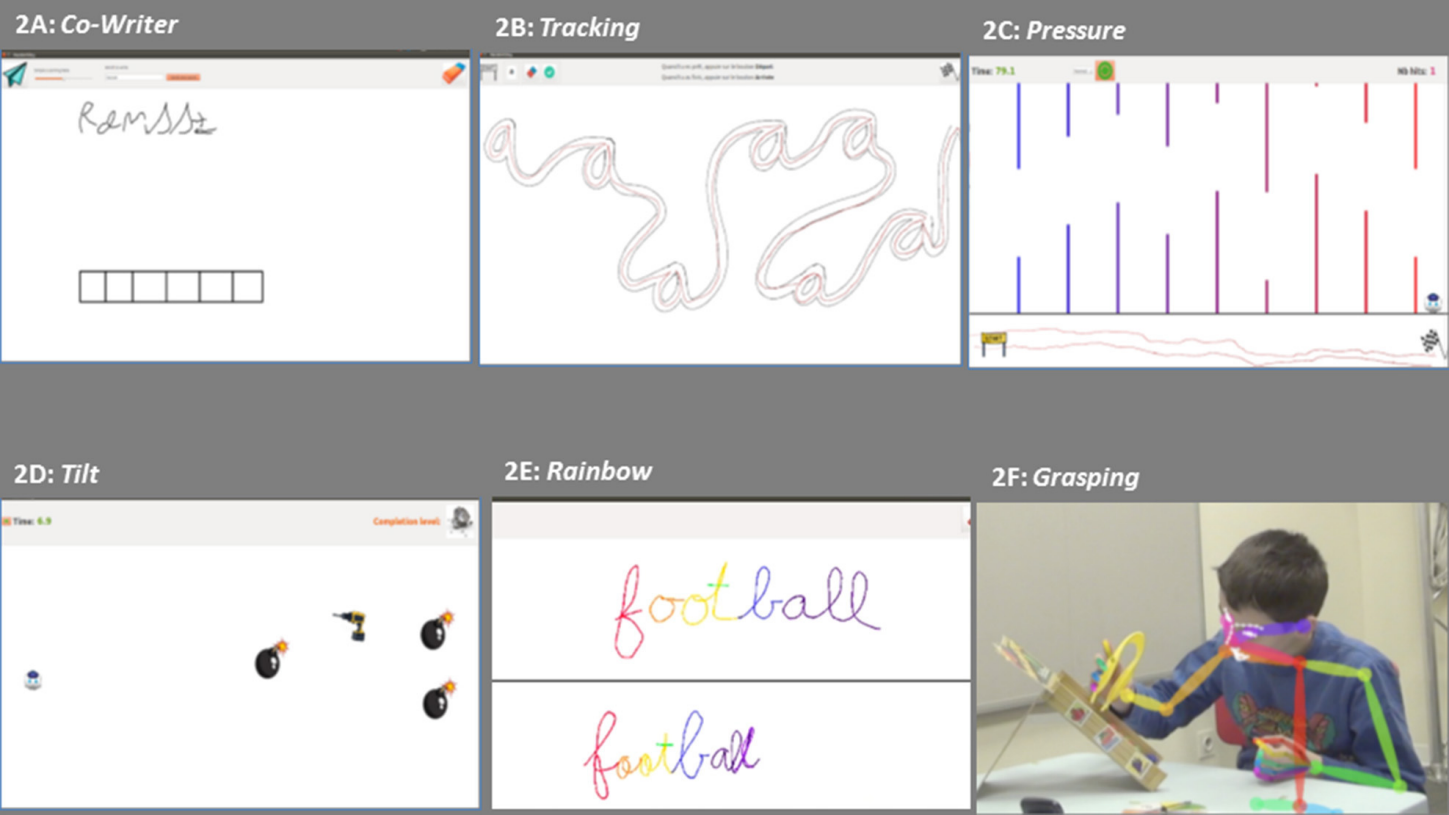
Screens from the tablet showing the different games used for handwriting training: **(A)** *Co-writer* activity; **(B)** Tracking; **(C)** Pressure; **(D)** Tilt; **(E)** Rainbow; and **(F)** Grasping.

The third component of the setup is the possibility to access a list of serious games computed in the tablet (Figure 2). The games evolved progressively based on the feedback from the child and the therapist. As we said previously, during the *Co-writer activity*, a robot writes a word in cursive with a bad handwriting. The goal is to have the child correct the robot by showing a “good handwriting.” The robot then learns from the child's handwriting and adapts its handwriting accordingly. The difficulty of the activity can be adapted by changing word length, frequency, and writing difficulty and the speed at which the robot “learns.”

The other games—*Dynamico*—were computed based on the fact that children with dysgraphia may be distinguished from typically developing children by characteristics related to speed, tilt, and pressure when writing (5, 23). We computed new activities to specifically train these skills. During *Tracking* (Figure 2B), the robot and the child are doing a track by following a layout in which we can find hidden letters. It is possible to change the level of difficulty of the activity by changing the hidden letter, the speed of the robot pursuing the player, and the width of the path. During *Pressure activity* (Figure 2C), the child controls a robot's head by moving the pen from left to right (between the sign *start* and the finish line) to control the *x* position of the robot while the *y* position is controlled by the amount of pressure the child applies between the pencil and the tablet. In order to avoid the obstacles within the game, the child needs to learn to control the amount of pressure he applies on the tablet. The difficulty of the activity can be adapted by changing the width of the aperture (the gap between bottom and upper wall) and the number of peaks. During the *Tilt activity* (Figure 2D), the child is using the pen like a joystick to control the robot head along the x and y axes. The goal of the activity is to capture the battery in order to recharge the robot while avoiding the bombs. It is possible to increase the level of difficulty by adding more bombs and diminishing their distance from the battery. Finally, the *Rainbow activity* allows making obvious the pauses during handwriting (Figure 2E). In a turn taking with the therapist that mirrors the *Co-writer* activity with Nao, the child writes alternatively on the tablet. First, the therapist writes a word (or a small text). Each time, there is a lift of the pen, the color of the ink changes. The child then needs to write the same word (or text) with the goal of reproducing the same color. If the color matches between the two words (one of child and one of therapist), it means that the child writes while performing pauses and liaisons in an optimal way.

The fourth component of the setup is the therapist who controls the rhythm of the therapy session, decides whether or not Nao gives feedbacks (e.g., “Come on, try again”) but can also participate in the gaming session when the child appears bored playing with Nao or asks to play the *grasping game* with the therapist. One after another, the therapist and the child need to grasp a fruit from a randomly chosen color and avoid the fall of all fruits. Finally, the setup also includes two 2D cameras to follow posture and face and offer specific metrics (Figure 2F).

### Experimental Design and Metrics

To assess longitudinally how R behaves during therapeutic sessions with the *Co-writer* setup, we monitored the sessions and registered several metrics, either clinical or digital, as both can be complementary to describe with more detail the motor difficulties of children with dysgraphia (5). We assessed (i) the acceptability and feasibility of the devises, software, and setup in a clinical setting using a qualitative approach with an observer listing all significant events and R's comments during sessions; (ii) how the handwriting improved according to digital metrics and the gold standard clinical testing of handwriting called BHK (35); (iii) how the posture of the child tracked with a 2D camera evolved through remediation, as it is known that children with dysgraphia show posture impairments during handwriting (2).

To assess writing, we collected BHK every five sessions. Each clinical BHK was randomly and blindly scored by two experts. We also computed several digital metrics to monitor R's progress within each game. Table 2 summarizes each metric per game. Finally, we recorded R's posture. The posture the child assumed during the handwriting sessions has been extracted and evaluated by analyzing high-definition videos (25 fps) of the BHK writing assessment (5 min writing of the same text). The camera was conveniently placed at a distance of 1.5 m from the front left of the child. Videos collected were analyzed frame-by-frame through the OpenPose library (36, 37) to extract a fine temporal evolution of the child skeleton. For each frame, the skeleton is composed of 94 key points in the (u,v) image space representing the position in the image of the body, of the hands, and of the facial landmarks of the child. Notably, for each extracted point, the OpenPose library exposes a confidence measure (p). The temporal evolution of the key points is then reconstructed using the frame rate of the camera. To ensure a reliable comparison between the metrics extracted from different videos captured on different days, the camera was fixed in its specific position, thanks to markers on the floor. Moreover, to minimize further possible errors, data were normalized among videos using the distance between the child's left eye and his left ear as a fixed, reliable reference, simple to compute.

**Table 2:** Digital metrics per *Co-Writer*/Dynamico activities.

**Activity**	**Possible metrics**	**Metrics shown in Figure 3**
*Co-writer*	Word length	During the cowriter activity, we tracked the average number of letters used in the word chosen with the child to teach the robot. Shorter words are easier than longer ones.
Tracking	Ratio between the number of points recorded outside the path and inside the path	During tracking activity, we tracked the success ratio and the child speed. A success corresponds to the fulfillment of the tracking task respecting the imposed path without the child leaving the path. Since it was a race with the robot, the cursor speed (here the head of the robot) was also tracked.
	Time required by the R to reach the end of the path (speed)	
Pressure	Level of difficulties to reach the maze	During the pressure activity, we tracked the difficulty and the time to reach the maze. The child was able to choose the difficulty of the exercise. An easier maze was a maze with more space between the obstacles and a more difficult one with a smaller space.
	Time required by the user to reach the end of the maze	
Tilt	Number of collisions with the bombs	In the tilt activity, due to the very high success rate, we tracked the time to finish the maze.
	Time spent before success	
Rainbow	Difference between the number of strokes recorded by the therapist and the child	

A metric indicating the quality of the child's posture was defined as the distance between his nose and his right hand since R was right-handed. This metric can be interpreted as a reflection of the body posture in the median anatomical plane. Small measures would indicate a head close to the table, while larger ones would suggest a better seat in his chair. Outliers were extracted and removed from the temporal evolution of the defined metric through a rolling window-based median filter and through the exclusion of aberrant samples lying outside ±2σ (standard deviation).

## Results

**Figure 3:**
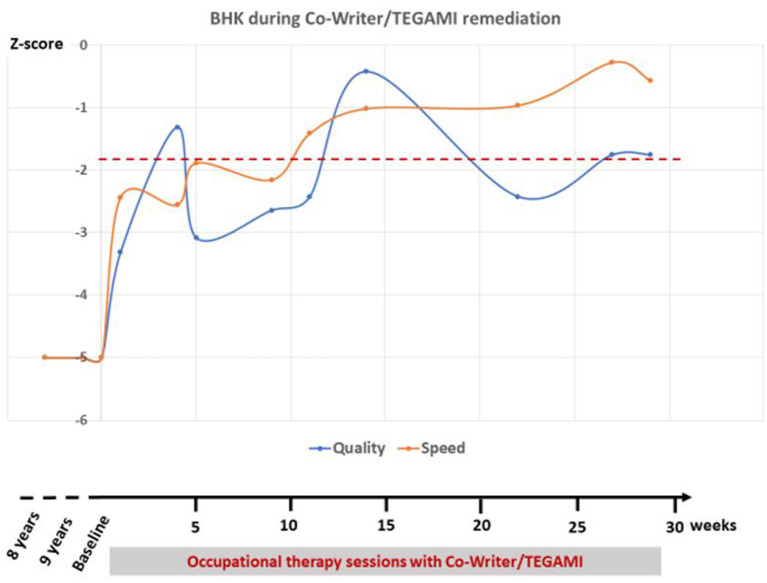
Clinical brave hand writing kinder (BHK) scores according to time during occupational therapy sessions with *Co-Writer*/Dynamico. The *z*-score shows how many standard deviation the handwriting quality/speed is compared to other children of the same gender and age. A child is diagnosed with dysgraphia when his score is below −1.8 (dotted red line). Of note, during the 30 weeks of treatment, R had 20 sessions in total because of remediation stops during vacation.

R immediately engaged with Nao. During the first sessions, he appeared to really believe in the scenario: he asked “*where does Nao come from?*,” “*Does he have siblings?*” He felt competitive and wanted to show him. Then, progressively, he understood that Nao “knew” how to write but was here to help him improve his handwriting: “*It is not the robot who learns, it is me*.” In the following sessions, he focused on gaming proposals, but Nao sometimes intertwined to support him and he smiled. During the 20 sessions of training, he tried all games, improved dramatically his behavior regarding schooling, and improved his handwriting. Figure 3 shows BHK scores according to time. Both writing quality and speed improved with time. As expected, when R tried to write faster, quality decreased for a brief period of time. At the end of the 20 sessions, around 500 min, he was now ready to go back to a regular school where he received special education (see video demo as presented at the International Conference on Robotics and Automation-ICRA 2020 conference, https://ieeetv.ieee.org/a-cowriter-robot-david-cohen).

**Figure 4:**
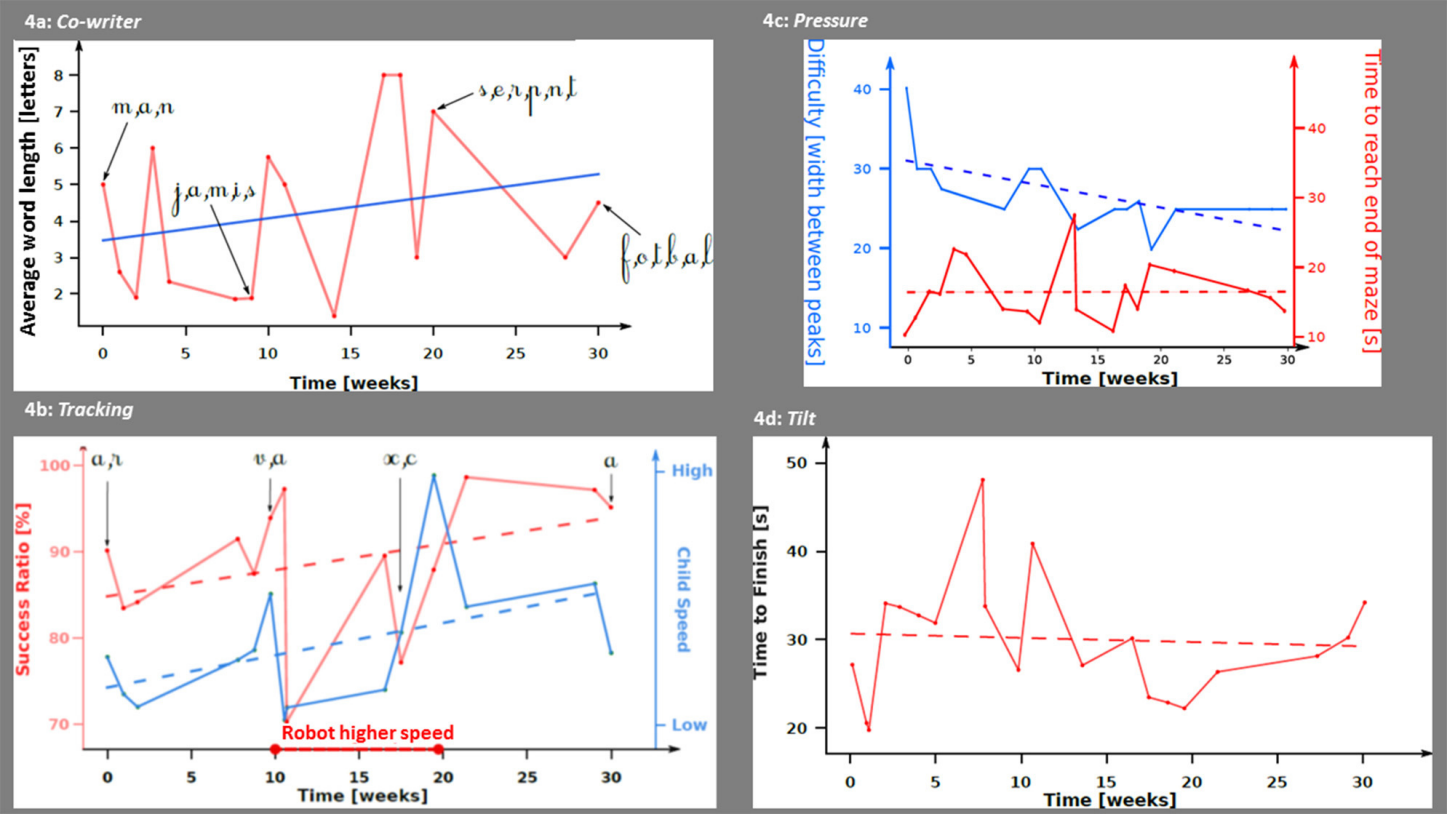
Digital metrics according to time during occupational therapy sessions with *Co-Writer*/Dynamico. **(a)** *Co-writer activity*. Average number of letters in the words written by R throughout the sessions. The blue line represents the evolution of the average number of letters computed with a linear regression. **(b)** *Tracking activity*. In red, the success ratio (ratio between the number of points recorded outside and inside the path); in blue, child's speed computed as the number of pixels per second. The dash lines represent the linear interpolations of both the success ratio and child's speed. During weeks 10 and 20, the robot's speed was increased by the therapists. **(c)** *Pressure activity*. In red, the time to reach the end of the maze; in blue, the width between the peaks (which is a proxy of the maze difficulty). The dash lines represent the linear interpolation of both the activity's difficulty and the time to reach the end of the maze. **(d)** *Tilt activity*. In red, the time to finish the activity; the dash lines represent the linear interpolation of the time to finish the activity.

Digital low-level metrics are summarized in Figure 4. During the *Co-Writer activity*, R was writing short words composed of simple letters like “man” at the beginning of the therapy while progressively writing longer and more complex words like “jamais” (never) at week 10 or “football” or “serpent” (snake) at week 30 (Figure 4a). During the *Tracking activity*, despite some fluctuations in the metrics that paralleled an increase of the robot's speed between week 10 and 30, we found an increase of both success ratio (which appears to be a proxy of precision) and R's handwriting speed (Figure 4b). During the *Pressure activity*, the time to reach the end of the maze (being a proxy of R's proficiency in the exercise) stayed relatively constant on average (around 15 s) despite a clear increase of the exercise difficulty (Figure 4c). This shows an improvement in the performance of R along the 30 weeks of therapy. During the *Tilt activity*, we found no decrease of the time R was taking to collect the five batteries (Figure 4d).

**Figure 5:**
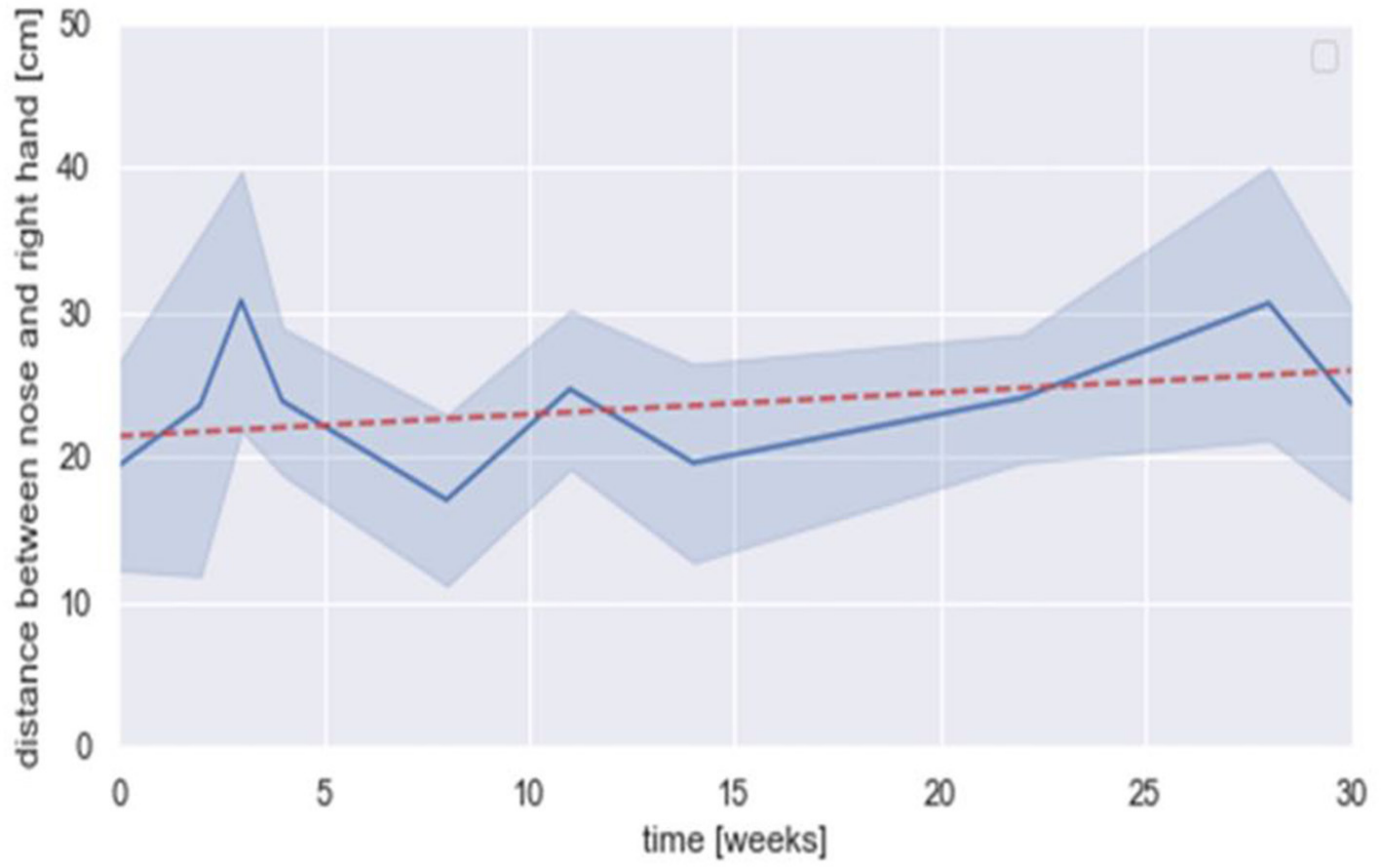
Distance between nose and right hand (cm) according to time during occupational therapy sessions with *Co-Writer*/Dynamico. Mean (in dark blue), standard deviation (in light blue), linear regression (in dot red) = [0.15 × +21.44] (*R*^2^ = 0.038).

Finally, R improved his posture during the sessions. As shown in Figure 5, the distance between nose and right hand increased from week 1 to 30: at the beginning of the treatment, R's head was close to the paper when he was writing with an average distance of 21 cm. At the end of the treatment, the average distance increased and the child was less bent on his writing sheet with a distance close to 30 cm.

## Discussion

We performed a long-term child–robot interaction to train the handwriting skills of a child with a complex NDD. The principle of the proposed treatment relies on multiple aims: relativizing and responsibility through a *protégé effect* scenario (27) employing a learning-by-teaching paradigm (30, 38); handwriting skills through a serious games platform proposing activities specifically aimed to exercise pressure, tilt, speed, and letter liaison controls (5, 23). In R's case, we observed a decrease of the avoidance behaviors, a better commitment, and an improvement of R's handwriting skills. We believe that a possible explanation for such observations would rely on the shift from the classical pen-and-paper rehabilitation paradigm to the presented scenario. Observations from future experimental studies involving larger samples would eventually confirm this hypothesis. The use of this longitudinal methodology has been made possible by the integration of several domains of expertise related to clinical science, development, computer science, and robotics (39). Interestingly, R's improvement of writing (Figure 1) followed the usual course of writing learning and automatization with steps: first of quality improvement then speed improvements (40–42). R also changed his posture during his writing progress as expected in learners who mature with writing (2).

However, the role of child–robot interaction and *Dynamico* may not be exclusive as the occupational therapist was still present during the sessions. Given the very experimental nature of our scenario, we wanted to ensure that an expert could follow the course of the sessions. In addition, during the beginning of the treatment, the design was iterative and patient-centered to seek a scenario development focused on the end user needs (26). This implies that we tried to integrate feedbacks that the occupational therapist provided after the first three sessions. The main innovations were to include the *Rainbow* game within *Dynamico* and to be directly involved in the sessions with the *Grasping activity*. We are aware that the role of the therapist in the presented scenario was not investigated. But we can speculate on its role from the anecdotal experience achieved from the presented case but also from two other contexts in which the identical setup was exploited: in a classical therapy setting with two occupational therapists in Lausanne (3) and in a classroom with children with ASD in Paris. Interestingly, even if the principles of fine motor skills and writing principles were similar, it seems that the strategies of occupational therapists could be quite different. With R, the occupational therapist had a developmental perspective, meaning that the child needed to master basic skills that would ultimately lead to mastering handwriting. She decided not to ask R to perform handwriting activities since R had refused to do so after 1 year of “pen-and-paper” occupational therapy with the same professional. In Lausanne, the occupational therapists were more intensive in their approach. The idea was to train writing, since, ultimately, that was the targeted goal. This diversity of approaches among occupational therapists is in line with dysgraphia literature review (see section *Introduction*) (2, 6, 8). In the context of the classroom, the teacher focused on how the children could think about their own strategy and performance, trying to praise them and guide them. While the therapists were very interventionist and wanted to tailor as much as possible the activities of the child to his/her needs, the teacher was interested in the use of the *Dynamico* device in semi-autonomy with the *Nao* robot. She said that a more autonomous system “would allow her to focus more on some children for other activities since their pedagogical goals could be different with different learning curves.”

Regarding serious games included in *Dynamico*, we proposed numerous scores related to features that were sensitive to changes and that paralleled clinical improvement. We hope in the future to compute a novel version of the serious game including tailored feedback based on these features to guide handwriting training and monitor the progresses of the child in a more autonomous way (43). These features congruent with the theoretical framework of digital phenotyping have the advantage to be motorized and thus easier to track (44). The usability of the setup was good for the therapist and the child, and the system was not invasive even after weeks of sessions, showing the promise of robotics in education (45). A formal evaluation of acceptability is planned with an improved version of the *Dynamico–Nao* setup in both occupational therapy sessions and in a classroom for children with special needs (15). Although several feature scores improved during R's treatment, it was not the case for the time to finish the tilt activity, which did not significantly decrease. One explanation is that during an automated handwriting, the tilt must be controlled and very stable (21). The change of tilt may not be a relevant feature for treatment assessment despite its relevance for classifying children with dysgraphia compared to typically developing children (23). An alternative hypothesis could be related to tilt activity in *Dynamico*. We wonder whether making feedbacks more explicit when the child touches an obstacle would help (sounds of explosion when he touches the bomb for instance). In addition, given the stability of the tilt during handwriting, we wonder whether a new activity training the stability of the tilt while changing the position of the pen would be of interest.

Beyond the acceptability and feasibility of this framework, we cannot generalize it or suggest some of its ingredients as a treatment of dysgraphia due to the limitation of a single case longitudinal methodology. Even if the failure of previous approaches to treat R's dysgraphia makes alternative hypotheses clinically unlikely (46), we cannot formally exclude a spontaneous resolution of dysgraphia. A randomized controlled trial with sufficient power will be necessary to make such claims of efficacy. Furthermore, it would be useful to assess the relative importance of either the complex system with a social robot or the writing tablet serious games alone. We also believe that using the serious games—*Dynamico*—implemented on much easier tablets (e.g., Ipad) would be of interest for scalability (43).

In this study, we performed analysis on low-level features that allowed giving real-time feedback during serious games directly based on position, pressure, and tilt. Future analysis should take into account more high-level features such as those described in Asselborn et al. (23) during BHK itself. They would allow to guide rehabilitation (1) by identifying the cluster of dysgraphia the child is in (5), by describing with more details the evolution of the child, since some exercises are more appropriate at the end than at the beginning of the rehabilitation (8).

The social interactions of the robot also had many limitations. We plan to endow it with more social skills to improve (1) the learning scenario, (2) the quality of the feedback, (3) metacognition and self-reflection of the child, and (4) motivation. Affective computing would be useful to assess the answers of the child after such behaviors. Robotics showed promising results in the field of special education, especially in the case of ASD, in which the children have interpersonal difficulties. Robots appear to be more predictable and reassuring for them (11, 13, 14, 16). A key aspect to be improved is also the general ergonomics of the system. While it allowed a rather fast improvement of writing in the case of R, the proposed experience was very heavy for clinical users due to time-consuming installation before starting a session, complex wiring, and unhandy interfaces. Besides the use of a stand-alone tablet (iPad®) to improve the user interface, we may also improve human robot interaction (HRI) smoothness with a more stable and social expressive robot (e.g., Qt robot).

We conclude that this longitudinal single case shows the feasibility and acceptability of the *Co-Writer* setup. Larger clinical studies are required to confirm that dysgraphia could benefit from this setup. We believe that implementation into the classroom as a regular educational proposal may also be a reasonable goal in particular if a version for stand-alone tablets may be computed.

## Data Availability Statement

The datasets presented in this article are not readily available because the data set cannot be shared publicly because of ethical restrictions. Data can't be de-identified, part of them have been acquired in a medical context and contain sensitive patient information. Requests to access the datasets should be directed to thomas.gargot@etud.univ-paris8.fr.

## Ethics Statement

Ethical review and approval was not required for the study on human participants in accordance with the local legislation and institutional requirements. Written informed consent to participate in this study was provided by the participants' legal guardian/next of kin. Written informed consent was obtained from the individual(s), and minor(s)' legal guardian/next of kin, for the publication of any potentially identifiable images or data included in this article.

## Author Contributions

TG designed the methodology, collected the data, analyzed the posture features, and wrote the first draft. TA developed iteratively the system based on the *Co-writer* project, analyzed the writing features, and wrote the first draft. IZ collected the data, run the occupational therapy sessions and the set-up, and tailored the use of the system to the R's needs: JB supervised R's medical management and the clinical assessment: PD, WJ, DA, and MC supervised the technical aspects both on software and robotics: DC coordinated the project, designed the methodology, and wrote the first draft: SA coordinated the project, designed the methodology, and analyzed the posture features. All authors approved the final version of the manuscript and gave specific inputs after the first draft.

## Conflict of Interest

The authors declare that the research was conducted in the absence of any commercial or financial relationships that could be construed as a potential conflict of interest.
